# The burden of injuries associated with accidents involving passengers carried in open bed pickup trucks - a review of 371 patients managed in a major trauma centre in South Africa

**DOI:** 10.1007/s00068-025-02857-z

**Published:** 2025-04-29

**Authors:** William Yeung, Victor Kong, Jonathan Ko, Reuben He, Jim Wang, Cynthia Cheung, Vasil Manchev, John Bruce, Grant Laing, Damian Clarke

**Affiliations:** 1 Department of Surgery, University of Auckland, Auckland, New Zealand; 2 Department of Surgery, Auckland City Hospital, Auckland, New Zealand; 3 Department of Surgery, University of KwaZulu Natal, Durban, South Africa; 4 Department of Surgery, University of the Witwatersrand, Johannesburg, South Africa; 5 Department of Surgery, Chris Hani Baragwanath Hospital, Johannesburg, South Africa

**Keywords:** Trauma, Pickup truck injuries, South Africa

## Abstract

**Introduction:**

Although the transportation of passengers in the open back area of pickup trucks is associated with significant risk of injury, this practice remains ubiquitous in South Africa. This study reviews the spectrum of injury and clinical outcome of these patients in a large city in South Africa The intention of the study is to highlight the inherent dangers associated with the practice and hence provide impetus to legislators and authorities to attempt to restrict and ultimately eliminate this practice.

**Materials and methods:**

This was a retrospective study conducted over a decade (Jan 2012-Dec 2023) at a major trauma centre in South Africa.

**Results:**

A total of 371 patients were included (male: 53%, mean age: 25 years). The mean Injury Severity Score (ISS) was 11. The most common anatomical region injured was head, followed by face and thorax. All patients underwent radiological investigations. 15% percent required operative intervention, with laparotomy and wound debridement being the most common. 9% required intensive care unit admission. The mean length of hospital stay was four days. The overall morbidity was 8%. The overall mortality was 4% and 71% of all mortalities were related to severe traumatic brain injury.

**Conclusions:**

Transporting passengers in the load area of a pickup truck is dangerous and results in preventable morbidity and mortality. Attention should be given in South Africa to developing legislation in order to prevent this activity and to enforce these rules once passed.

## Introduction

Road traffic accidents (RTA) remain a global health crisis with over 1.3 million people dying from RTAs and many more suffering from debilitating injuries each year [1]. Although all countries struggle with this issue, low or middle income countries (LMIC), such as South Africa, suffer disproportionately from this issue [2]. An estimated mortality rate of 20.7 per 100 000 is reported for South Africa in 2022 compared to a mortality rate of 7 per 100 000 in Europe [3]. 

RTAs are preventable provided there is effective legislation coupled with the enforcement of traffic regulations and road and vehicle safety features [4]. Reducing the burden of RTA related injury relies on improved safety engineering and on enforced safety legislation [5]. To be effective, these interventions must be informed by reliable data.

A source of this data is from clinical audit of injury patterns associated with specific types of road traffic related injuries. Pickup trucks, which are colloquially referred to as ‘*bakkies*’ in South Africa, have an extended open cargo bed designed for transporting a load. This makes the bakkie an attractive and popular vehicle class and over 100,000 units annually are sold in the country [6]. As of January 2024, bakkies make up 22% of all total self-propelled vehicles in South Africa [7]. While intended to safely carry cargo, bakkies in South Africa are frequently used to transport passengers and livestock in the cargo bed. This is strictly prohibited in countries with more rigorous safety codes [8]. Despite several lethal accidents involving the transportation of passengers in the open bed of bakkies, there has been minimal sustained public health attention focused on this issue in South Africa [9]. 

This study attempts to address this deficit by reviewing all patients admitted to a major trauma centre follow accidents from ‘back of the bakkie’. The aim was to categorise the spectrum of injuries seen and establish a profile of injury pattern associated with this specific mechanism. It is hoped that this information will inform public health advocacy surrounding the need for more stringent legislation in regard to this ubiquitous practice, and that this increased awareness, both locally and internationally, will generate political pressure to force authorities and legislators to begin addressing this issue.

## Materials and methods

### Clinical setting

The Pietermaritzburg Metropolitan Trauma Service (PMTS) based at Grey’s Hospital in Pietermaritzburg, South Africa, is the tertiary trauma service to Pietermaritzburg and its surrounding catchments. The PMTS is an academic trauma centre with both undergraduate and postgraduate trainees exposed to over 4000 trauma admissions annually. The electronic registry at our institution, called Hybrid Electronic Medical Registry (HEMR), was established in 2012 and captures all patients admitted to our institution. Patient data from HEMR is not de-identified.

### The study

Data from January 2012 to December 2022 was retrieved from the HEMR. All patients who suffered injury that were documented as to related to accidents where the passenger was being transported in the back of a bakkie were identified and reviewed. This cohort was then further analysed on their demographics, injuries sustained, radiological investigations and subsequent operation(s) (if applicable), length of hospital stay, complications and outcomes. Statistical analysis was done with Microsoft Excel. Ethics approval for the maintenance of our electronic trauma registry and for conducting this study was formally approved by the Biomedical Research Ethics Committee of the University of Kwa Zulu Natal (Ethics approval number: BCA 221/13).

## Results

### Overview

During the ten-year study period, a total of 371 patients were admitted to the PMTS with injuries related to being passengers in the back of a bakkie. There were 195 males (53%) and the mean age of the cohort was 25 years (SD 15). The mean Injury Severity Score (ISS) was 11. The median value of physiological variables on admission was summarised in Table 1.

**Table 1:** Mean admission physiology of 371 patients

Median Physiological Variables	(Interquartile Range)
Respiratory Rate (breaths per minute)	18 (16–20)
Heart Rate (beats per minute)	90 (76–105)
Systolic Blood Pressure (mmHg^†^)	117 (106–130)
Shock Index	0.8 (0.6–0.9)
Glasgow Coma Score (GCS)	15 (13–15)
pH	7.40 (7.36–7.44)
Lactate (mmol/L^‡^)	1.5 (0.9–2.7)

^†^ millimetres of mercury

^‡^ millimoles per litre

### Spectrum of injury

The spectrum of injury according to anatomical region was summarised in Table 2. The head was the most common injured region (75%), followed by face and the thorax. Table 3 summarises the injuries sustained to each anatomical region.

**Table 2:** Spectrum of injuries by anatomical region

Spectrum of Injuries by Anatomical Region	Number of Injuries
Head	278
Face	120
Neck	34
Thorax	103
Abdomen	92
Urogenital	4
Pelvis	61
Upper Extremity	94
Lower Extremity	86

**Table 3:** Spectrum of injury sustained for each anatomical region

Spectrum of Injury Sustained for each Anatomical Region	Number of Injuries
**Head** Soft Tissue Musculoskeletal Focal Diffuse Neurology	**496** 186 101 156 49 4
**Face** Soft Tissue Musculoskeletal Nerve	**153** 90 62 1
**Neck** Soft Tissue Musculoskeletal Vascular Nerve	**32** 5 21 3 3
**Thorax** Soft Tissue Musculoskeletal Pulmonary Cardiac Vascular Nerve	**166** 41 56 61 2 2 4
**Abdomen** Soft Tissue Musculoskeletal Vascular Organ Gastrointestinal Tract Urogenital	**102** 42 20 2 21 5 12
**Pelvis** Soft Tissue Musculoskeletal Perineal	**72** 32 36 4
**Extremities** Soft Tissue Musculoskeletal Vascular Nerve	**200** 143 55 1 1

### Management of injuries

All patients underwent radiological investigations. The most common modalities were plain radiography and CT scan. The radiological investigations conducted are summarised in Table 4.

**Table 4:** Imaging modalities used in cohort of 371 patients

Imaging Modalities Used	*N* = 1 348
**Plain Radiographs** Chest Abdomen Pelvis Extremities Cervical Spine Skull	**655** 219 39 130 99 112 66
**Ultrasound** Focussed Assessment with sonography for Trauma (FAST) Formal	**24** 14 10
**CT** Chest Abdomen Pelvis Spine Head Maxillofacial Full Body (PAN)	**632** 34 92 40 149 228 14 75
**MRI** Head Spine	**4** 1 3
**Others** Angiogram Cystogram	**33** 10 23

### Operative procedures

A total of 57 (15%) patients required an operation, of which 40 (70%) required a single operation, 15 (26%) two operations and two (4%) required three operations. A total of 76 operative procedures were performed. The most common operation was laparotomy (18%) followed by debridement of wounds (17%) and open reduction and internal fixation (13%). The spectrum of operations were summarised in Table 5.

**Table 5:** Summary of operations performed on 57 patients

Operations	*N* = 76
Laparotomy	14
Debridement	13
Open reduction internal fixation	10
Decompressive craniotomy	9
Bladder Repair	6
Laparoscopy	5
Burr Hole	4
Examination under anesthesia	2
Other	13

### Clinical outcomes

9% (33/371) of patients required intensive care unit (ICU) admission. The mean length of hospital stay was four days. Thirty patients (8%) developed one or more complication during their hospital stay. These morbidities are summarised in Table 6. The overall mortality was 4%. Over two thirds (71%, 10/14) of all mortalities were due to severe traumatic brain injury. Two patients suffered aspiration pneumonia and both developed refractory sepsis and multi organ failure. A single patient sustained a fatal venous thromboembolism and a single patient exsanguinated form a pelvic fracture.

**Table 6:** Summary of morbidities among 30 patients during their hospital stay

*Morbidities*	*N* = 35
Respiratory	6
Renal	3
Gastrointestinal	3
Neurological	5
Wound Sepsis	6
Other	12

### Trend over time

**Fig. 1:**
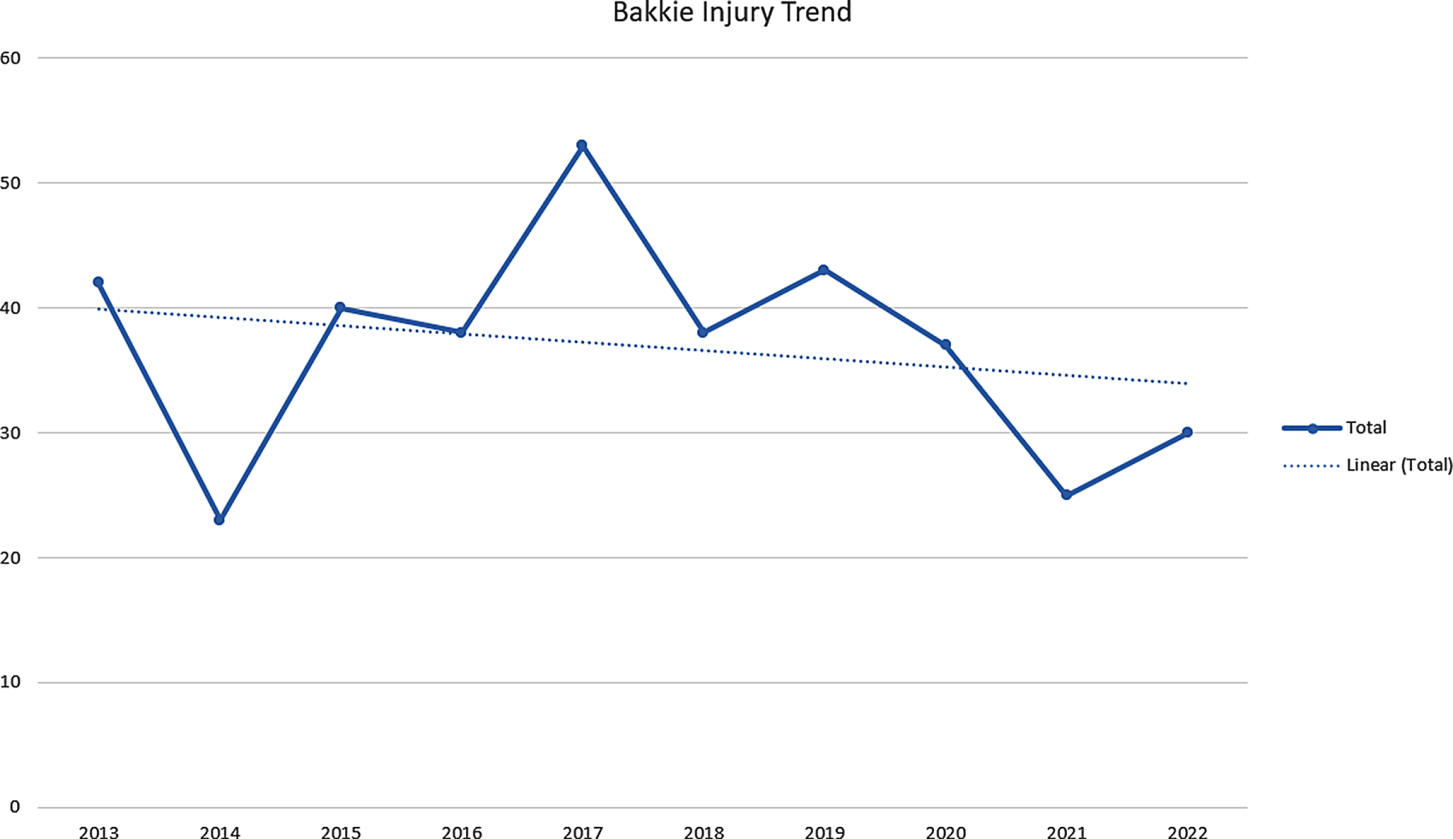
Trend in the number of patients with bakkie injury managed per year

**Fig. 2:**
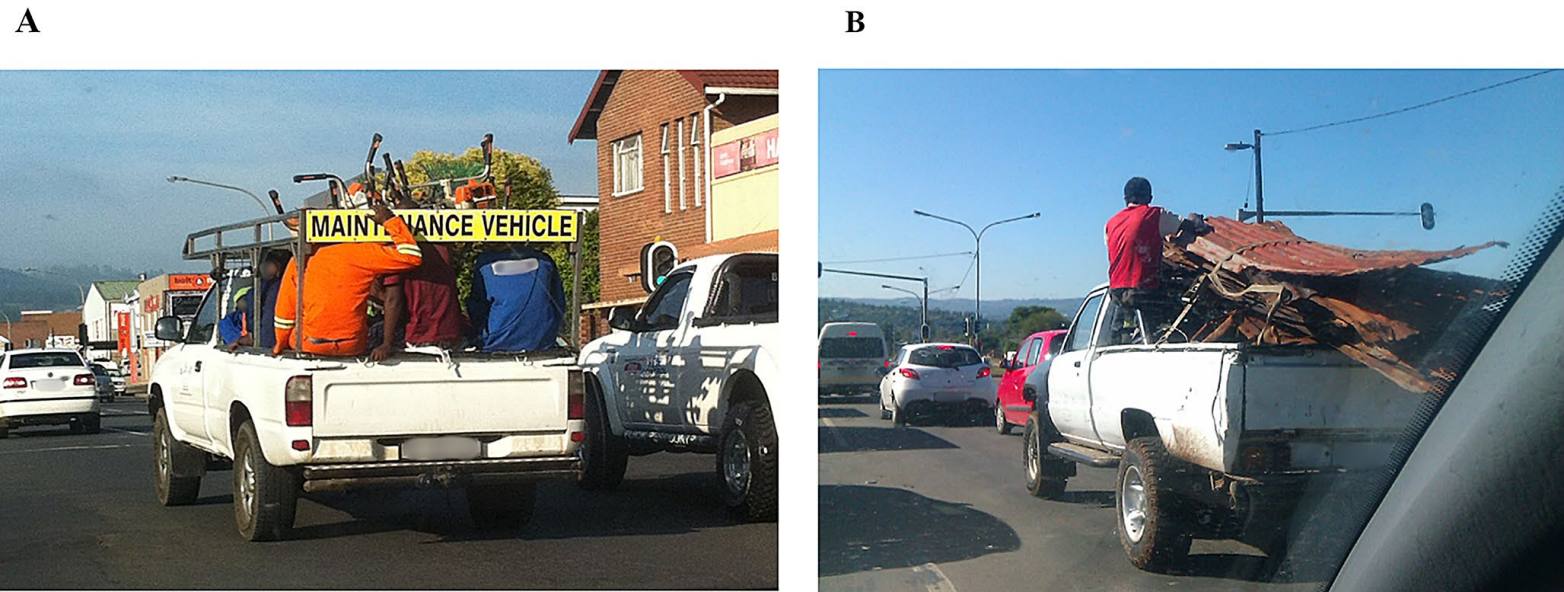
Pickup trucks transporting people in the open bed cargo area. (Authors personal collection)

During the 10 year study period, 2014 had the lowest number of admissions while 2017 had the highest. There was an overall trend of slight reduction in the total number of patients over the period. The authors believe that this downward trend may have been skewed due to effects of COVID lockdown reducing the number of vehicles on the road and therefore reducing bakkie related injuries in 2020–2022. This is summarised in Figs. 1 and 2.

## Discussion

Bakkies are not intended for the transportation of passengers. The load area has no seats, no restraining devices and no roof. These vehicles can travel at high speed and unrestrained passengers in the open bed are highly vulnerable. In South Africa, the use of these vehicles to transport passengers is ubiquitous. Often lift clubs are formed to transport passengers. This is particularly common for commuting to work and for transporting children to and from school [10]. Transporting passengers in the open cargo area of a bakkie is associated with substantial risk. There is a significant risk of being ejected from the vehicle in the event of a crash. This is in stark contrast to the situation for restrained passengers travelling in a closed roof type vehicle. Being ejected from a vehicle remains a serious mechanism of trauma frequently associated with a diverse injury pattern.

Studies from North America provide insight into this road safety related issue. Bucklew et al. and Anderson et al. have convincingly argued that passengers carried in the open cargo area of bakkies are at increased risk of morbidity and mortality, as they have limited protection in an event of a crash [11, 12]. These authors documented a high incidence of head injury and noted a significant mortality rate. The data from our study echoes these findings, with traumatic brain injury (TBI) being the most common injury. Over 70% of all mortality in our study was secondary to severe TBI. Passengers involved in RTAs while travelling in the back of a bakkie also have a significant risk of sustaining a devastating neurological injury such as quadriplegia or paraplegia [13]. These injuries result in significant financial costs to the healthcare system, including 873 days in hospital, 70 days in ICU, and 17 operations [9].

The economic impact of these injuries is difficult to quantify, but is significant. A Canadian study estimated that 352 injured patients cost Canadian society over five-million Canadian dollars in terms of health care costs and loss of income [14]. In 2015, the total cost of RTAs in South Africa was estimated to be 3.4% of South Africa’s GDP [15]. This is similar to reports from other LMIC’s [16]. Once again, these figure are likely to be conservative estimates [17]. RTAs tend to disproportionately affect young patients who are breadwinners, and injuries to patients from lower socioeconomic groups tend to be more severe and debilitating [18, 19]. 

Our study demonstrates that accidents related to transportation of passengers in the back of a bakkie result in diverse injuries, a significant proportion of which require operative intervention. These injuries consume significant healthcare resources and negatively impact on the patient’s ability to earn an income. Given that LMIC’s disproportionately sustain 92% of fatal RTAs, interventions are required to reduce this burden [16]. 

The current rules related to the use of bakkies as a means of passenger transport are opaque and the legislation which exists is seldom enforced in South Africa. This is in spite of the fact that legislation and road safety interventions have been effective in reducing RTA related morbidity and mortality in LMIC’s [20, 21]. Scholars have theorised that this may be a result of informal institutions, such as values and behaviours of road users, undermining the effectiveness of formal institutions, such as the law [22]. This suggests that South Africa may require a tailored approach to road safety rather than a one size fits all model derived from high income countries.

Although RTAs are preventable, legislation, enforcement and safety design must be evidence-based. This study focuses specifically on a mechanism which is associated with a significant mortality and morbidity rate and which impacts on young people. Whilst constituting a subset of the overall burden of RTA related morbidity and mortality, back of bakkie injuries are relatively easy to target with appropriate legislation and enforcement. Public health policy is always searching for a “bang for the buck type” solution, where a relatively inexpensive and socially well tolerated intervention leads to major improvements in outcomes and safety. This is similar to experience with the enforcement of safety helmets for motor bike riders, where a relatively inexpensive and easily enforceable intervention significantly reduced serious head injuries by 88% [23]. Similarly, interventions such as seatbelt use, random breath testing and speed limits have also shown to be cost effective interventions for road traffic injuries in LMIC [24]. There is no need to invest massively in safety infrastructure, or to increase public spending, to eliminate this problem.

Although this study is limited by being restricted to a single centre, it is hoped that it will highlight the problem and stimulate larger comprehensive national audits. These further studies must encompass a wider catchment area to further delineate and inform the economic impact of bakkie related RTAs. These studies will also have to include mortuary data and police reports to provide a comprehensive overview. The transportation of passengers in the back of a bakkie is a public health concern and efforts must be directed at strengthening the current legislation and enforcing these regulations to reduce the frequency of these injuries.

## Conclusions

Transporting passengers in the load area of a pickup truck is dangerous and results in significant preventable morbidity and mortality. South Africa should develop legislation to prevent this activity. Appropriate enforcement of these regulations once passed, is essential.

## Data Availability

No datasets were generated or analysed during the current study.
